# Posttraumatic stress disorder in people who use drugs: syringe services program utilization, treatment need, and preferences for onsite mental health care

**DOI:** 10.1186/s12954-024-01019-5

**Published:** 2024-06-01

**Authors:** Teresa López-Castro, Nancy Sohler, Lindsey Riback, Gina Bravo, Eric Ohlendorf, Megan Ghiroli, Aaron D. Fox

**Affiliations:** 1 Department of Psychology, The City College of New York, City University of New York, 160 Convent Avenue, New York, NY 10031 USA; 2 The City University of New York School of Medicine, 160 Convent Avenue, New York, NY 10031 USA; 3 Albert Einstein College of Medicine, 1300 Morris Park Ave, Bronx, NY 10461 USA; 4 Montefiore Medical Center, 111 East 210th Street, Bronx, NY 10467 USA

**Keywords:** Substance use disorders, Psychiatric comorbidity, Harm reduction, Integrated posttraumatic stress disorder and substance use disorder care, Opioid-related overdose

## Abstract

**Background:**

Syringe services programs (SSPs) are critical healthcare access points for people with opioid use disorder (OUD) who face treatment utilization barriers. Co-locating care for common psychiatric comorbidities, like posttraumatic stress disorder (PTSD), at SSPs may reduce harms and enhance the health of individuals with OUD. To guide the development of onsite psychiatric care at SSPs, we collected quantitative survey data on the prevalence of PTSD, drug use patterns, treatment experiences associated with a probable PTSD diagnosis, and attitudes regarding onsite PTSD care in a convenience sample of registered SSP clients in New York City.

**Methods:**

Study participants were administered the PTSD Checklist for the *DSM-5* (PCL-5) and asked about sociodemographic characteristics, current drug use, OUD and PTSD treatment histories, and desire for future SSP services using a structured interview. Probable PTSD diagnosis was defined as a PCL-5 score *≥* 31.

**Results:**

Of the 139 participants surveyed, 138 experienced at least one potentially traumatic event and were included in the present analysis. The sample was primarily male (*n* = 108, 78.3%), of Hispanic or Latinx ethnicity (*n* = 76, 55.1%), and middle-aged (*M* = 45.0 years, *SD* = 10.6). The mean PCL-5 score was 35.2 (*SD* = 21.0) and 79 participants (57.2%) had a probable PTSD diagnosis. We documented frequent SSP utilization, significant unmet PTSD treatment need, and high interest in onsite PTSD treatment.

**Conclusions:**

Study findings point to the ubiquity of PTSD in people with OUD who visit SSPs, large gaps in PTSD care, and the potential for harm reduction settings like SSPs to reach people underserved by the healthcare system who have co-occurring OUD and PTSD.

## Background

Posttraumatic stress disorder (PTSD) develops after experiencing or witnessing a traumatic event such as an assault, rape, or disaster. Symptoms include re-experiencing the traumatic event, avoiding trauma reminders, negative alterations in mood and thought, and heightened arousal and reactivity [1]. PTSD is one of the most common psychiatric comorbidities in people who use drugs (PWUD) with prevalence estimates ranging between 21% and 40% [2–5]. From a risk environment framework [6], this disproportionately high PTSD rate can be understood as a product of the social situations and structures PWUD inhabit (e.g. drug economy involvement, housing policies, and policing practices that increase the likelihood of exposures to potentially traumatic events [7]). PTSD among PWUD independently confers a two-fold risk of experiencing a non-fatal overdose [4] and predicts a higher risk of drug and sexual practices linked to HIV acquisition and transmission [8].

PTSD frequently co-occurs with opioid use disorder (OUD) and their comorbidity intensifies the burden of each disorder. In comparison with those living with only OUD, PTSD is associated with greater severity of OUD symptoms, polydrug use, higher likelihood of comorbid depression and attempted suicide, and poorer physical and mental health [9]. It is also well-documented that PTSD complicates the process of engaging in care for OUD [10, 11]. While PWUD living with comorbid PTSD report greater interest in OUD treatment than PWUD not living with PTSD, the frequency of engaging in OUD treatment does not differ between these groups [12, 13]. This suggests that PWUD living with PTSD may recognize their need for OUD care but face unique barriers to treatment. These barriers might arise from PTSD symptoms themselves, such as intrusive memories, avoidance, and emotion dysregulation, which together may make navigating complex health systems, tolerating long waiting times, and enduring stigma particularly difficult [2, 14]. Engagement in the conventional health care system for OUD care is, therefore, challenging for individuals with comorbid PTSD and OUD.

Structural barriers also prevent PWUD from accessing traditional sites for PTSD care. Identifying and accessing specialists can be challenging, availability of specialized PTSD care remains limited outside of the Veterans Administration system [15], and PWUDs are commonly subjected to stigma in their interactions with the healthcare system [16, 17]. One way to simplify access to PTSD care for this population is to integrate its provision with OUD care. Concurrent and integrated care models for PTSD and substance use disorders have demonstrated effectiveness [18], yet widespread adoption remains very limited [15]. Co-locating PTSD care within OUD treatment programs or prescribing buprenorphine treatment at a specialized mental health clinic are examples of integrated care. However, these models have been developed and tested in traditional outpatient and academic hospital centers, which are difficult for many PWUDs to access [19, 20]. Thus, alternative models of care delivery outside of specialized healthcare settings will likely be necessary to reach PWUD who live with PTSD.

Syringe services programs (SSPs) represent promising, novel venues for integrated OUD and PTSD treatment. SSPs effectively reach PWUD who may infrequently use conventional sources of healthcare. SSPs engage PWUD by working from a harm reduction framework that actively counteracts stigma, makes minimal demands on clients to access care, and prioritizes building client trust [21–24]. SSPs are widespread throughout the U.S. with more than 400 operating in 43 states, Washington, DC, and Puerto Rico [25]. In addition to providing sterile syringes and injection equipment, SSPs offer a range of health and social services that include vaccinations, HIV/HCV testing and care, and linkage to substance use disorder treatment [26]. With the 2020 lifting of an in-person examination requirement for buprenorphine initiation, 24% of SSPs currently offer onsite buprenorphine initiation via telehealth [27]. Single-site trials of this low-threshold approach to OUD care have shown preliminary effectiveness [28, 29]. The feasibility of locating specialized, integrated, “low-threshold” psychiatric care at SSPs, however, is unknown.

Despite the lack of effectiveness data, about 15% of SSPs in the U.S. reported offering onsite mental health counseling in 2020 [30]. To most effectively expand this strategy to integrate evidence-based PTSD care with low-threshold OUD services at SSPs, we sought to answer several questions. First, what is the prevalence of PTSD in the SSP setting? Second, how much OUD and PTSD care do SSP clients already receive at traditional treatment sites or onsite at SSPs? Finally, would integrated OUD and mental health care at SSPs be desired by SSP clients? We explored these questions among registered SSP clients recruited in New York City (NYC). Research has consistently shown that there are higher rates of psychiatric diagnoses, including PTSD, in PWUD than in the general population [3]. We thus expected to find elevated rates of trauma and PTSD and significant unmet PTSD treatment needs in this sample of SSP clients. Additionally, we hypothesized that a provisional PTSD diagnosis would be predictive of lower OUD, PTSD, and SSP services utilization [2, 13, 31]. Our question about attitudes toward mental health care onsite at SSPs was exploratory.

## Method*s*

### Participants and procedure

We recruited a convenience sample from three SSPs in NYC between June 2021 and March 2022. Recruitment occurred in-person at the SSPs and through SSP staff referrals. Eligibility criteria were being a registered SSP client, being over 18 years of age, and self-report of an OUD diagnosis. A research team member conducted a brief screening interview to establish eligibility. All SSP clients encountered by the research team at the SSPs who met eligibility criteria were invited to participate in the study. Those who provided oral consent were administered a 30-minute survey by a trained research staff member. Surveys were completed in person in a private office at the SSP or via telephone in either English or Spanish. Participants received $20 in cash as compensation. The Einstein College of Medicine Institutional Review Board approved the study.

### Measures

#### Trauma exposure and PTSD symptomatology

We used the Life Events Checklist for the *DSM*-*5*-Extended Version to assess lifetime exposure to potentially traumatic events (LEC-5-EV; [32]). The LEC-5-EV presents 16 event types and, for each type, asks respondents to identify the type of exposure (e.g., personally exposed, witnessed exposure, heard about exposure) and the timing of the exposure (within the last 6 months), and then asks respondents to provide brief characteristics regarding the worst event. LEC-5-EV responses were used to establish the presence of a traumatic stressor (PTSD Criterion A).

PTSD symptoms were measured using the PTSD Checklist for *DSM-5* (PCL-5; [33]), a widely used, psychometrically validated, self-report questionnaire [34]. The PCL-5 consists of 20 items that align with the 20 *DSM-5* PTSD symptoms and measures symptom severity in the past month. Respondents rate each item on a five-point Likert-like scale (0 to 4). When there is an endorsement of a potentially traumatic event, a PCL-5 score of 31 or greater has been demonstrated to be a robust indicator of probable PTSD diagnosis [35]. Thus, we categorized people who endorsed a potentially traumatic event as having none to low PTSD symptoms (PCL-5 < 31), and having probable PTSD (PCL-5 *≥* 31).

### Drug use

Participants self-reported their alcohol and drug use using an adapted version of the Drug/Alcohol Use section of the Addiction Severity Index [36]. Data included the number of days that they used the following drugs, respectively, in the last 30 days: alcohol, amphetamines, benzodiazepines, cannabis, cocaine, heroin/fentanyl, methadone, buprenorphine, other opioids/analgesics, and synthetic cannabis or K2. For each drug with at least one day of use reported, participants then identified their usual route of administration and, for methadone, buprenorphine, opiates/analgesics, benzodiazepines, and amphetamines, participants reported whether the drug was prescribed to them. We collected information on lifetime opioid-related overdose, which was defined as “being unresponsive or unable to be woken up, waking up in a hospital or ambulance, collapsing or losing consciousness, having difficulty breathing, or having blue skin due to heroin, fentanyl, or another opioid.”

### Prior OUD treatment, PTSD treatment, and unmet treatment needs

We asked participants, “In your lifetime, what opioid use disorder treatments have you used?” Participants then reported whether they had received each of seven different types of OUD treatment, respectively (yes/no). Treatment options were “Detoxification” (i.e., medically managed withdrawal services), “Outpatient program with groups or one-on-one counseling,” “Inpatient or residential program,” “Methadone treatment,” “Buprenorphine or Suboxone treatment,” “Naltrexone treatment,” and “Mutual aid groups (Alcoholics Anonymous and/or Narcotics Anonymous).” If participants answered yes to receiving a treatment, they were then asked the number of episodes in which they had participated in that treatment.

We collected information about mental health treatment history using an adapted version of the Psychiatric Status section of the Addiction Severity Index [36]. We added the question, “Has a health professional ever diagnosed you with a psychological or emotional problem?” and if so, “What was/were the diagnosis(es)”? Data collected included the number of times participants were treated for any psychological or emotional problems, the setting of care (e.g. hospital/inpatient and outpatient), how many treatment episodes included PTSD treatment, and recency of PTSD-related care (e.g. in the past six months or longer).

### SSP utilization and future service preferences

We asked participants about their utilization of SSPs and the various services offered at SSPs. The first question asked about the frequency of visiting the SSP in the past month. The next set of questions asked about the past 30-day utilization of specific services, including individual mental health counseling, group mental health counseling, psychiatric medication visits, medications for OUD, and OUD counseling. The last set of questions asked about services that participants would like to receive onsite at an SSP in the future.

### Other information

Data were also collected regarding age, gender (male, female, transgender, or gender non-conforming), ethnicity (Hispanic/Latinx or not), and health insurance status.

### Data analysis

We first identified participants who endorsed a potentially traumatic event in the LEC-5 and described the traumatic events reported for those with probable PTSD as measured by the PCL-5. We then categorized individuals with potentially traumatic events based on their report of past-month PTSD symptoms (PCL-5 *≥* 31 [probable PTSD)] versus PCL-5 < 31 [low PTSD symptoms]). We compared participants who had probable PTSD with those who had low PTSD symptoms on sociodemographic characteristics, drug use behaviors, OUD and mental health treatment histories, SSP utilization histories, and desired SSP services. Data were analyzed using IBM SPSS Statistics, version 29 [37]. We report means and standard deviations for continuous variables and proportions for categorical variables and assess the statistical significance of the difference between the two groups on each measure using t-tests and chi-square tests.

## Results

A total 139 participants completed the study. One hundred and thirty-eight people reported at least one potentially traumatic event in their lifetime and were included in the analyses that follow. The mean PCL-5 score in the sample was 35.2 (*SD* = 21.0). Seventy-nine people (57.2%) had a PCL-5 score of *≥* 31 and 59 people (42.8%) had a PCL-5 score of < 31. Table 1 summarizes the event types of lifetime trauma exposure of those with a PCL-5 score *≥* 31 (probable PTSD).

**Table 1:** Trauma event types endorsed by 79 registered SSP clients with a probable PTSD diagnosis in New York City

Potentially traumatic event types endorsed on the LEC-5 (happened to me)		*n* (% of 79)
Physical assault		58 (73.4)
Assault with a weapon		47 (59.5)
Transportation accident		45 (57.0)
Severe human suffering		38 (48.1)
Serious accident		29 (36.7)
Natural disaster		33 (41.8)
Other very stressful event or experience		32 (40.5)
Life-threatening illness or injury		28 (35.4)
Witnessing a sudden violent death		26 (32.9)
Sexual assault		25 (31.6)
Unwanted/uncomfortable sexual experience (other than sexual assault)		24 (30.4)
Fire or explosion		21 (26.6)
Witnessing a sudden accidental death		17 (21.5)
Combat or war-zone exposure		14 (17.7)
Serious injury/harm/death you caused to other(s)		16 (20.3)
Toxic substance exposure		11 (13.9)
Captivity		3 (3.8)

***Note***. LEC-5: Life Events Checklist for ***DSM-5***; PTSD: posttraumatic stress disorder; probable PTSD diagnosis = PCL-5 ≥ 31; SSP: syringe services program

The mean age of the sample was 45.0 years (*SD* = 10.6). Most of the participants were male at birth (*n* = 108, 78.3%). Over half reported being of Hispanic or Latinx ethnicity (*n* = 76, 55.1%). Black (*n* = 49, 35.5%) and White (*n* = 49, 35.5%) were the most commonly reported racial categories. The majority reported having health insurance (*n* = 129, 93.5%). The most commonly used drugs were heroin or fentanyl (*M* = 17.83 [*SD* = 13.1] days per month), cocaine (*M* = 16.0 [*SD* = 13.3] days per month), and alcohol (*M* = 5.3 [*SD* = 9.7] days per month).

Comparisons between those with PCL-5 score *≥* 31 and those who had a PCL-5 score < 31 demonstrated that significantly more participants with probable PTSD reported any mental health diagnosis (*n* = 66, 83.5% vs. *n* = 35, 59.3%, χ^2^ [1] = 10.10, *p* < 0.01), a known PTSD diagnosis (*n* = 32, 40.5% vs. *n* = 10, 16.9%, χ^2^ [1] = 8.85, *p* < 0.01), and having ever received PTSD treatment (*n* = 38, 48.1% vs. *n* = 18, 30.5%, χ^2^ [1]^1^ = 4.34, *p* = 0.04). Participants with probable PTSD were also more likely to report prior opioid overdose (*n* = 52, 85.8% vs. *n* = 29, 49.2%, χ^2^ [1] = 3.87, *p* < 0.05). Table 2 shows demographic characteristics and drug use behaviors.

**Table 2:** Demographic characteristics, drug use behaviors, and past OUD and mental health treatment of 138 registered SSP clients with a history of OUD and trauma exposure in New York City by PTSD symptom severity

Sociodemographic characteristics	Probable PTSD *n* (% of 79)	Low PTSD symptoms *n* (% of 59)	*p*-value
*M *(*SD*) age	45.6 (11.0)	44.2 (10.2)	0.44
Hispanic or Latinx	40 (50.6)	36 (61.0)	0.23
Any health insurance	72 (91.1)	57 (96.6)	0.20
Male gender	59 (74.7)	49 (83.1)	0.24
*Drug use characteristics*			
*M* (*SD*) days/past 30 days			
Alcohol	5.8 (10.1)	4.7 ( 9.1)	0.51
Cocaine	16.7 (13.5)	15.0 (13.1)	0.46
Heroin/fentanyl	17.9 (13.4)	17.7 (12.8)	0.92
Other opioids	3.1 ( 7.6)	1.4 ( 4.8)	0.13
Benzodiazepine	4.6 ( 9.0)	5.3 ( 9.7)	0.65
Injected drugs, ever	50 (63.3)	46 (78.3)	0.06
Opioid-related overdose, ever	52 (65.8)	29 (49.2)	< 0.05*
*Prior OUD treatment*			
Detoxification	63 (79.7)	49 (83.1)	0.62
Counseling	53 (67.1)	42 (71.2)	0.61
Inpatient/residential	62 (78.5)	39 (66.1)	0.10
Methadone (*n* = 134)	55 (71.4)	42 (73.7)	0.77
Buprenorphine	33 (41.8)	26 (44.1)	0.79
Naltrexone	4 (5.1)	3 ( 5.1)	< 1.00
Narcotics or Alcohol Anonymous	60 (75.9)	33 (55.9)	0.01*
*Prior mental health treatment*			
Diagnosis, any	66 (83.5)	35 (59.3)	0.001**
Diagnosis, PTSD	32 (40.5)	10 (16.9)	0.003**
Outpatient	49 (62.0)	26 (44.1)	0.04*
Inpatient	44 (55.7)	21 (35.6)	0.02*
PTSD treatment, ever	38 (48.1)	18 (30.5)	0.04*
PTSD treatment, past 6 months	17 (21.5)	7 (11.9)	0.14

***Note***. SSP: syringe services program; OUD: opioid use disorder; PTSD: posttraumatic stress disorder; probable PTSD = PCL-5 *≥* 31; low PTSD symptoms = PCL-5 < 31; * = ***p*** < 0.05; ** = ***p*** < 0.01

Table 3 compares the utilization of SSP services and future desire for onsite SSP services by level of PTSD symptom severity. Most (59.5%) participants with probable PTSD visited the SSPs at least daily and this was not statistically significantly different from participants with low PTSD symptoms (χ^2^ [1] = 3.23, *p* = 0.07). A minority of participants in each group had received onsite mental health or OUD treatment services at SSPs, but the majority of participants in each group desired for these services to be available at the SSPs.

**Table 3:** SSP service utilization and preferences of 138 registered SSP clients with a history of OUD and trauma exposure in New York City by PTSD symptom severity

Utilization of SSP Services	Probable PTSD *n* (% of 79)	Low PTSD symptoms *n* (% or 59)	*p*-value
Frequency, visit at least daily	47 (59.5)	26 (44.1)	0.07
*OUD treatment*			
Counseling	21 (26.6)	15 (25.4)	0.88
OUD medications	12 (15.2)	12 (20.3)	0.43
*Mental health treatment*			
Counseling, individual	31 (39.2)	15 (25.4)	0.09
Counseling, group	24 (30.4)	10 (16.9)	0.07
Medication	13 (16.5)	6 (10.2)	0.29
*Desire for onsite SSP services*			
* OUD treatment*			
Counseling	60 (75.9)	38 (64.4)	0.14
OUD medications	53 (67.1)	42 (71.2)	0.61
* Mental health treatment*			
Counseling, individual	65 (82.3)	43 (72.9)	0.19
Counseling, group	57 (72.2)	37 (62.7)	0.24
Medication	55 (69.6)	33 (55.9)	0.10

***Note***. SSP: syringe services program; OUD: opioid use disorder; PTSD: posttraumatic stress disorder; probable PTSD = PCL-5 *≥* 31; low PTSD symptoms = PCL-5 < 31

## Discussion

SSPs effectively reach and engage PWUD [38] who are frequently marginalized from traditional healthcare outlets [39, 40]. SSPs may further serve as linchpin venues for improving the health of individuals with OUD by addressing psychiatric comorbidities like PTSD. In our NYC sample of SSP clients with a history of OUD and current drug use, trauma exposure was pervasive (99.2%). We found a high prevalence of probable PTSD (57.2%). Additionally, there was a high burden of PTSD symptoms in the sample as a whole, even among those who did not meet the criteria for probable PTSD. We also documented gaps in PTSD-specific care and participant interest in receiving PTSD treatment delivered onsite at SSPs.

The magnitude of trauma exposure and elevated rate of probable PTSD in our study were expected, indicative of the cumulative burden among individuals served by SSPs. Other research has shown that, compared with PWUD who do not use SSPs, those accessing SSPs carry greater structural- and individual-level vulnerabilities (e.g., housing instability, injection drug use practices, co-occurring stimulant use, street-based income, and sex-work involvement) that increase the likelihood of violence and health-related harms [5, 41, 42]. However, the prevalence of probable PTSD in our sample (57.2%) was even greater than has been reported in prior work with SSP-referred methadone clients. In a sample of newly-enrolled methadone clients referred from a Baltimore SSP, Kidorf et al. [3] found that 21% met *DSM-IV* criteria for PTSD. This lower rate may reflect sampling differences between studies. If PTSD symptoms interfere with treatment seeking, then SSP clients who successfully engage in methadone treatment may have fewer PTSD symptoms than clients who do not.

There were three other main findings. Firstly, a significant proportion of SSP clients had unmet PTSD treatment needs. Among people with a probable PTSD diagnosis, 40.5% reported a prior PTSD diagnosis and only 48.1% reported PTSD treatment in the past. As expected, the endorsement of prior PTSD treatment by those with PTSD symptoms that fell below the diagnostic cut-off was significantly lower than for those in the probable PTSD group. The lack of treatment in those with low PTSD symptoms warrants concern because, despite not meeting diagnostic criteria, subthreshold levels of PTSD carry substantial functional impairments [43, 44]. In light of evidence that untreated PTSD can stall efforts to treat substance use disorders [10, 13, 45], untreated PTSD observed in this study stands as a modifiable barrier to OUD treatment engagement in this high-need population and merits critical attention.

Second and relatedly, in our study, probable PTSD was associated with lifetime endorsement of having had an opioid-related overdose. This result mirrors the findings of a recent study conducted with a large Vancouver cohort of PWUD [4] and earlier work with PWUD in rural Appalachia [46]. Our study further supports the possibility that PTSD-related distress may privilege avoidant coping, reinforce opioid use, and impede adaptive help-seeking behaviors [47, 48]. This is an especially pernicious effect for individuals with OUD who already face significant drug-related harms.

Lastly, people with probable PTSD reported receiving services at the SSPs and they appeared to desire additional mental health and OUD treatment services there. Other data demonstrate that SSP clients trust and have supportive relationships with SSP staff, which contrasts with their experiences seeking care in traditional healthcare settings [16, 19, 21]. Indeed, most in our sample reported using SSP-based healthcare services. These findings run counter to the perception that marginalized PWUD are resistant to or do not want treatment [17]. Efforts to seek and engage fully in treatment can be impaired by PTSD symptoms, but provider- and systems-level barriers to PTSD care may also limit service usage. Our findings suggest the strong possibility that onsite PTSD care at SSPs will be acceptable to its clients—if delivered in a fashion consonant with the person-centered, destigmatizing ethos of harm reduction organizations [23, 49].

Delivering PTSD care in low-threshold settings like SSPs would likely involve multidisciplinary coordination and require increased resources, but prior evidence suggests that it can be done. PTSD treatments have demonstrated efficacy when delivered in a variety of low-resource settings where exposure to adversity and violence may be chronic and ongoing [50–52]. The availability of providers with specialization in the evidence-based treatment of PTSD may be another limitation. However, gold-standard PTSD care has been effectively adapted for delivery through telehealth [53]. Integrating pharmacotherapy with harm reduction behavioral approaches such as those espoused by SSPs has been studied in the context of low-threshold, supportive housing. Randomized controlled trials of naltrexone in combination with harm reduction psychotherapy have shown success with individuals who are experiencing chronic homelessness and live with severe alcohol use disorder [54, 55]. Thus, developing low-threshold models of PTSD and OUD care appears to be warranted.

Our findings carry several implications related to mental health service provision to PWUD at SSPs. From a therapeutic perspective, our results confirm the importance of adopting a trauma-informed approach when designing and implementing health services to PWUD. Beyond an understanding of trauma-specific interventions, a trauma-informed approach recognizes the impact of trauma upon the individual and takes into consideration these experiences in the present context of care [56]. The harm reduction principles that guide SSP service provision already dovetail substantially with a trauma-informed approach that prioritizes client’s safety, transparency, collaboration, and empowerment [57]. On a public health level, our results suggest that SSPs serve a population of PWUD with substantial PTSD burden who are interested in receiving mental health care and willing to doing so in the SSP context. Together, these findings underscore the potential role that SSPs can play in bridging the mental health treatment gap in PWUD [58] and the benefits of increasing the availability of SSPs in the US equipped to deliver onsite psychiatric services. As first steps, barriers currently limiting the funding for SSPs and their equitable coverage in the US [59] must be addressed as well as the training and educating of mental health care professionals in harm reduction principles. There can sometimes be cultural differences between health care providers and harm reduction practitioners, therefore integrating psychiatric services into SSPs will require substantial cultural humility [60]. Rigorous research is critical; to the best of our knowledge, no research or evaluation of onsite SSP screening and treatment for PTSD have been conducted.

The strengths of our study include our ability to reach PWUD, a validated PTSD screening instrument, and trained research staff to assist in survey completion. Our study also has limitations worthy of note. Firstly, our sample size was constrained by physical distancing precautions implemented during the COVID-19 pandemic. As a convenience sample from a large metropolitan area, our findings may not generalize to other types of SSP settings such as smaller-city and rural-serving SSPs. Relatedly, obtaining a true estimate of PTSD prevalence at SSPs would involve more systematic sampling procedures to accurately account for subpopulations in the SSP setting potentially underrepresented here (e.g., women, transgender individuals, and people with housing insecurity). Because this study focused exclusively on PTSD symptomatology we are unable to shed light on the presence and associations of other psychiatric disorders known to be elevated in the population who use SSPs [3, 61]. Moreover, PTSD is also highly comorbid with a range of mood, anxiety, and personality disorders [62]. Future work is tasked with systematically documenting the full extent of psychiatric burden, psychosocial needs, and unique preferences of people who utilize SSP services.

## Conclusion

Amid a historic rise in U.S. drug overdoses [63] and the persistent underutilization of evidence-based OUD treatments [58], novel access points are critically needed to engage and retain individuals with OUD in treatment. One such possibility involves offering integrated OUD and psychiatric care in venues trusted and frequented by PWUD. Our study documented high rates of PTSD in individuals with self-reported OUD who currently utilized SSPs in NYC, unmet need for PTSD treatment, and strong interest in SSP-based PTSD care. These findings support the design of co-located PTSD services that are responsive to the needs, preferences, and harm reduction context of SSPs and the communities of PWUD they serve.

## Data Availability

The dataset generated and analyzed during the current study is not publicly available to protect the privacy of respondents but is available from the corresponding author on reasonable request.

## Abbreviations

PTSD: Posttraumatic stress disorder
PWUD: People who use drugs
OUD: Opioid use disorder
SSPs: Syringe services programs
NYC: New York City
LEC-5-EV: Life Events Checklist for *DSM**-**5*-Extended Version
PCL-5: PTSD Checklist for *DSM-5*

## References

[1] American Psychiatric Association (2013). Diagnostic and statistical manual of mental disorders.

[2] Goytan A, Lee W, Dong H, Hayashi K, Milloy MJ, Kerr T (2021). The impact of PSTD on service access among people who use drugs in Vancouver, Canada. Subst Abuse Treat Prev Policy.

[3] Kidorf M, Solazzo S, Yan H, Brooner RK (2018). Psychiatric and substance use comorbidity in treatment-seeking injection opioid users referred from syringe exchange. J Dual Diagnosis.

[4] Lee WK, Hayashi K, DeBeck K, Milloy MJS, Grant C, Wood E (2020). Association between posttraumatic stress disorder and nonfatal drug overdose. Psychol Trauma: Theory Res Pract Policy.

[5] Mitra S, Lee W, Hayashi K, Boyd J, Milloy MJ, Dong H (2021). A gender comparative analysis of post-traumatic stress disorder among a community-based cohort of people who use drugs in Vancouver, Canada. Addict Behav.

[6] Rhodes T (2009). Risk environments and drug harms: a social science for harm reduction approach. Int J Drug Policy.

[7] Forbes D, Lockwood E, Phelps A, Wade D, Creamer M, Bryant RA (2013). Trauma at the hands of another: distinguishing PTSD patterns following intimate and nonintimate interpersonal and noninterpersonal trauma in a nationally representative sample. J Clin Psychiatry.

[8] Plotzker RE, Metzger DS, Holmes WC (2007). Childhood sexual and physical abuse histories, PTSD, depression, and HIV risk outcomes in women injection drug users: a potential mediating pathway. Am J Addictions.

[9] Mills KL, Lynskey M, Teesson M, Ross J, Darke S (2005). Post-traumatic stress disorder among people with heroin dependence in the Australian Treatment Outcome Study (ATOS): prevalence and correlates. Drug Alcohol Depend.

[10] Ecker AH, Hundt N. Posttraumatic stress disorder in opioid agonist therapy: a review. Psychol Trauma. 2017. 10.1037/tra0000312 28758767

[11] Mills KL, Teesson M, Ross J, Darke S (2007). The impact of post-traumatic stress disorder on treatment outcomes for heroin dependence. Addiction.

[12] Kidorf M, King VL, Pierce J, Kolodner K, Brooner RK (2011). Benefits of concurrent syringe exchange and substance abuse treatment participation. J Subst Abuse Treat.

[13] Peirce JM, Brooner RK, King VL, Kidorf MS (2016). Effect of traumatic event reexposure and PTSD on substance use disorder treatment response. Drug Alcohol Depend.

[14] King VL, Brooner RK, Peirce J, Kolodner K, Kidorf M (2014). Challenges and outcomes of parallel care for patients with co-occurring psychiatric disorder in methadone maintenance treatment. J Dual Diagnosis.

[15] Killeen TK, Back SE, Brady KT (2015). Implementation of integrated therapies for comorbid post-traumatic stress disorder and substance use disorders in community substance abuse treatment programs. Drug Alcohol Rev.

[16] Muncan B, Walters SM, Ezell J, Ompad DC (2020). They look at us like junkies: influences of drug use stigma on the healthcare engagement of people who inject drugs in New York City. Harm Reduct J.

[17] Fong C, Mateu-Gelabert P, Ciervo C, Eckhardt B, Aponte-Melendez Y, Kapadia S (2021). Medical provider stigma experienced by people who use drugs (MPS-PWUD): development and validation of a scale among people who currently inject drugs in New York City. Drug Alcohol Depend.

[18] Hien DA, Morgan-López AA, Saavedra LM, Ruglass LM, Ye A, López-Castro T (2023). Project Harmony: a meta-analysis with individual patient data on behavioral and pharmacologic trials for comorbid posttraumatic stress and alcohol or other drug use disorders. American Journal of Psychiatry.

[19] Paquette CE, Syvertsen JL, Pollini RA (2018). Stigma at every turn: health services experiences among people who inject drugs. Int J Drug Policy.

[20] Wang L, Panagiotoglou D, Min JE, DeBeck K, Milloy MJ, Kerr T (2016). Inability to access health and social services associated with mental health among people who inject drugs in a Canadian setting. Drug Alcohol Depend.

[21] Biancarelli DL, Biello KB, Childs E, Drainoni M, Salhaney P, Edeza A (2019). Strategies used by people who inject drugs to avoid stigma in healthcare settings. Drug Alcohol Depend.

[22] Lago RR, Peter E, Bógus CM (2017). Harm reduction and tensions in trust and distrust in a Mental Health Service: a qualitative approach. Subst Abuse Treat Prev Policy.

[23] Macneil J, Pauly B (2011). Needle exchange as a safe haven in an unsafe world. Drug Alcohol Rev.

[24] Treloar C, Rance J, Yates K, Mao L (2016). Trust and people who inject drugs: the perspectives of clients and staff of Needle Syringe Programs. Int J Drug Policy.

[25] NASEN. North American Syringe Exchange Network | NASEN [Internet]. [cited 2023 Apr 10]. Available from: https://nasen.org.

[26] Centers for Disease Control. Syringe Services Programs (SSPs) | CDC [Internet]. 2021 [cited 2023 Apr 10]. https://www.cdc.gov/ssp/index.html.

[27] Lambdin BH, Bluthenthal RN, Tookes HE, Wenger L, Morris T, LaKosky P (2022). Buprenorphine implementation at syringe service programs following waiver of the Ryan Haight Act in the United States. Drug Alcohol Depend.

[28] Bachhuber MA, Thompson C, Prybylowski A, Benitez J, Mazzella S, Barclay D (2018). Description and outcomes of a buprenorphine maintenance treatment program integrated within Prevention Point Philadelphia, an urban syringe exchange program. Substance Abuse.

[29] Hood JE, Banta-Green CJ, Duchin JS, Breuner J, Dell W, Finegood B (2020). Engaging an unstably housed population with low-barrier buprenorphine treatment at a syringe services program: lessons learned from Seattle, Washington. Substance Abuse.

[30] Behrends CN, Lu X, Corry GJ, LaKosky P, Prohaska SM, Glick SN (2022). Harm reduction and health services provided by syringe services programs in 2019 and subsequent impact of COVID-19 on services in 2020. Drug Alcohol Depend.

[31] Peirce JM, Brooner RK, Kolodner K, Schacht RL, Kidorf MS (2013). Prospective effects of traumatic event re-exposure and post-traumatic stress disorder in syringe exchange participants. Addiction.

[32] Weathers FW, Blake DD, Schnurr PP, Kaloupek DG, Marx BP, Keane TM (2013). The life events checklist for DSM–5 (LEC-5).

[33] Weathers FW, Litz BT, Keane TM, Palmieri P, Marx BP, Schnurr PP (2013). The PTSD Checklist for DSM–5 (PCL-5).

[34] Lee DJ, Weathers FW, Thompson-Hollands J, Sloan DM, Marx BP (2022). Concordance in PTSD symptom change between DSM-5 versions of the Clinician-administered PTSD scale (CAPS-5) and PTSD checklist (PCL-5). Psychol Assess.

[35] Blevins CA, Weathers FW, Davis MT, Witte TK, Domino JL (2015). The posttraumatic stress disorder checklist for DSM-5 (PCL-5): development and initial psychometric evaluation. J Trauma Stress.

[36] McClellan AT, Luborsky L, Woody G, O’Brien C (1980). An Improved Diagnostic evaluation instrument for substance abuse patients: the Addiction Severity Index. J Nerv Ment Dis.

[37] IBM Corp (2023). IBM SPSS statistics for Windows, Version 29.0.2.0. Armonk.

[38] Des Jarlais DC, McKnight C, Goldblatt C, Purchase D (2009). Doing harm reduction better: syringe exchange in the United States. Addiction.

[39] Kreek MJ (2011). Extreme marginalization: addiction and other mental health disorders, stigma, and imprisonment. Ann N Y Acad Sci.

[40] Richardson LA, Long C, DeBeck K, Nguyen P, Milloy MJS, Wood E (2015). Socioeconomic marginalisation in the structural production of vulnerability to violence among people who use illicit drugs. J Epidemiol Community Health.

[41] Ti L, Richardson L, DeBeck K, Nguyen P, Montaner J, Wood E (2014). The impact of engagement in street-based income generation activities on stimulant drug use cessation among people who inject drugs. Drug Alcohol Depend.

[42] Wood E, Lloyd-Smith E, Li K, Strathdee SA, Small W, Tyndall MW (2007). Frequent needle exchange use and HIV incidence in Vancouver, Canada. Am J Med.

[43] Morgan-López AA, Killeen TK, Saavedra LM, Hien DA, Fitzpatrick S, Ruglass LM (2020). Crossover between diagnostic and empirical categorizations of full and subthreshold PTSD. J Affect Disord.

[44] Norman SB, Stein MB, Davidson JRT (2007). Profiling posttraumatic functional impairment. J Nerv Ment Dis.

[45] Norman SB, Tate SR, Anderson KG, Brown SA (2007). Do trauma history and PTSD symptoms influence addiction relapse context?. Drug Alcohol Depend.

[46] Havens JR, Oser CB, Knudsen HK, Lofwall M, Stoops WW, Walsh SL (2011). Individual and network factors associated with non-fatal overdose among rural Appalachian drug users. Drug Alcohol Depend.

[47] Ehring T, Quack D (2010). Emotion regulation difficulties in trauma survivors: the role of trauma type and PTSD symptom severity. Behav Ther.

[48] Tull MT, Barrett HM, McMillan ES, Roemer L (2007). A preliminary investigation of the relationship between emotion regulation difficulties and posttraumatic stress symptoms. Behav Ther.

[49] Allen ST, Grieb SM, O’Rourke A, Yoder R, Planchet E, White RH (2019). Understanding the public health consequences of suspending a rural syringe services program: a qualitative study of the experiences of people who inject drugs. Harm Reduct J.

[50] Bass JK, Annan J, McIvor Murray S, Kaysen D, Griffiths S, Cetinoglu T (2013). Controlled trial of psychotherapy for Congolese survivors of sexual violence. N Engl J Med.

[51] Pearson CR, Kaysen D, Huh D, Bedard-Gilligan M (2019). Randomized control trial of culturally adapted Cognitive Processing Therapy for PTSD substance misuse and HIV sexual risk behavior for Native American women. AIDS Behav.

[52] Weiss WM, Murray LK, Zangana GAS, Mahmooth Z, Kaysen D, Dorsey S (2015). Community-based mental health treatments for survivors of torture and militant attacks in Southern Iraq: a randomized control trial. BMC Psychiatry.

[53] Fortney JC, Bauer AM, Cerimele JM, Pyne JM, Pfeiffer PN, Heagerty P (2021). A pragmatic randomized comparative effectiveness trial of tele-integrated care and tele-referral care for treating complex psychiatric disorders in primary care. JAMA Psychiatry.

[54] Collins SE, Duncan MH, Saxon AJ, Taylor EM, Mayberry N, Merrill JO (2021). Combining behavioral harm-reduction treatment and extended-release naltrexone for people experiencing homelessness and alcohol use disorder in the USA: a randomised clinical trial. Lancet Psychiatry.

[55] Collins SE, Clifasefi SL, Nelson LA, Stanton J, Goldstein SC, Taylor EM (2019). Randomized controlled trial of harm reduction treatment for alcohol (HaRT-A) for people experiencing homelessness and alcohol use disorder. Int J Drug Policy.

[56] Huang LN, Flatow R, Biggs T, Afayee S, Smith K, Clark T et al. SAMHSA’s Concept of Truama and Guidance for a Trauma-Informed Approach [Internet]. Substance Abuse and Mental Health Services Administration (SAMHSA); 2014 Jul [cited 2024 Apr 23]. https://archive.hshsl.umaryland.edu/handle/10713/18559.

[57] Substance Abuse and Mental Health Services Administration. SAMHSA Harm Reduction Framework. Center for Substance Abuse Prevention, Substance Abuse and Mental Health Services Administration; 2023.

[58] Substance Abuse and Mental Health Services Administration (2022). Key substance use and mental health indicators in the United States: results from the 2021 National Survey on Drug Use and Health (HHS Publication No. PEP22-07-01-005.

[59] Fernández-Viña MH, Prood NE, Herpolsheimer A, Waimberg J, Burris S (2020). State laws governing syringe services programs and participant syringe possession, 2014–2019. Public Health Rep.

[60] Heller D, McCoy K, Cunningham C (2004). An invisible barrier to integrating HIV primary care with harm reduction services: philosophical clashes between the harm reduction and medical models. Public Health Rep.

[61] Mackesy-Amiti ME, Donenberg GR, Ouellet LJ (2012). Prevalence of psychiatric disorders among young injection drug users. Drug Alcohol Depend.

[62] Goldstein RB, Smith SM, Chou SP, Saha TD, Jung J, Zhang H (2016). The epidemiology of DSM-5 posttraumatic stress disorder in the United States: results from the National Epidemiologic Survey on Alcohol and Related Conditions-III. Soc Psychiatry Psychiatr Epidemiol.

[63] Ahmad F, Cisewski J, Rossen L, Sutton P. Provisional drug overdose death counts. National Center for Health Statistics; 2023.

